# Data-driven prediction of *in situ* CO_2_ foam strength for enhanced oil recovery and carbon sequestration†

**DOI:** 10.1039/d2ra05841c

**Published:** 2022-12-14

**Authors:** Javad Iskandarov, George S. Fanourgakis, Shehzad Ahmed, Waleed Alameri, George E. Froudakis, Georgios N. Karanikolos

**Affiliations:** Department of Chemical Engineering, Khalifa University P. O. Box 127788 Abu Dhabi UAE georgios.karanikolos@ku.ac.ae; Research and Innovation Center on CO_2_ and H_2_ (RICH), Khalifa University P. O. Box 127788 Abu Dhabi UAE; Department of Chemistry, University of Crete Voutes Campus Heraklion GR-70013 Crete Greece frudakis@uoc.gr; Department of Petroleum Engineering, Khalifa University P. O. Box 127788 Abu Dhabi UAE; Center for Catalysis and Separations (CeCaS), Khalifa University P. O. Box 127788 Abu Dhabi UAE; Department of Chemical Engineering, University of Patras 26504 Patras Greece; Laboratory of Quantum and Computational Chemistry, Department of Chemistry, Aristotle University of Thessaloniki 54124 Thessaloniki Greece

## Abstract

Carbon dioxide foam injection is a promising enhanced oil recovery (EOR) method, being at the same time an efficient carbon storage technology. The strength of CO_2_ foam under reservoir conditions plays a crucial role in predicting the EOR and sequestration performance, yet, controlling the strength of the foam is challenging due to the complex physics of foams and their sensitivity to operational conditions and reservoir parameters. Data-driven approaches for complex fluids such as foams can be an alternative method to the time-consuming experimental and conventional modeling techniques, which often fail to accurately describe the effect of all important related parameters. In this study, machine learning (ML) models were constructed to predict the oil-free CO_2_ foam apparent viscosity in the bulk phase and sandstone formations. Based on previous experimental data on various operational and reservoir conditions, predictive models were developed by employing six ML algorithms. Among the applied algorithms, neural network algorithms provided the most precise predictions for bulk and porous media. The established models were then used to compute the critical foam quality under different conditions and determine the maximum apparent foam viscosity, effectively controlling CO_2_ mobility to co-optimize EOR and CO_2_ sequestration.

## Abstract 2

Data-driven approaches for foams can be a time- and data-efficient alternative method to experimental and conventional modeling techniques.

## Graphical abstract

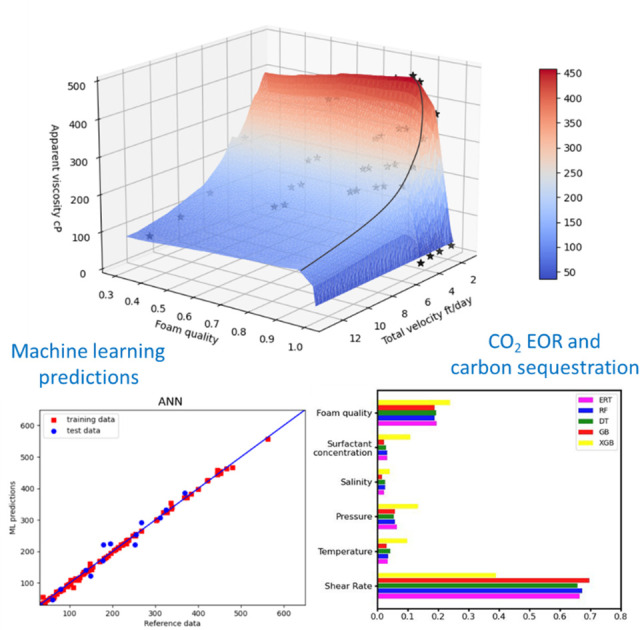

## 1. Introduction

Due to emerging environmental impact, greenhouse gas (GHG) emissions have been the global community's focus over the last few decades. Although the world is gradually moving away from fossil fuel usage, a complete transition may take decades. Since CO_2_ is one of the most significant contributors to climate change, its capture before its release into the atmosphere significantly benefits the environment.^1,2^ Therefore, new, more efficient carbon capture technologies are desired to minimize the amount of GHG in the atmosphere. In addition, long-term storage and utilization of post-exhaust gases following their capture are necessary. One of the few large-scale carbon capture, utilization, and storage (CCUS) technologies is the enhanced oil recovery (EOR) method.^3–6^ EOR processes aim to increase oil extraction by injecting a replacement media. Among the media used in gas EOR, CO_2_ is the most attractive, particularly in the USA, where natural CO_2_ sources are abundant. Several researchers argue that CO_2_ EOR negatively impacts GHG mitigation since it is used to produce fossil fuels, that lead to more CO_2_.^7^ However, recent studies show that CO_2_ EOR can result in a negative net carbon emission when considering the tertiary oil recovery process and the consumption of used hydrocarbons.^8^ In other words, the stored amount of CO_2_ is higher than the emitted one during the downstream and upstream stages. In addition, during the oil extraction, most of the injected CO_2_ gas remains stored in the reservoir^8^ achieving this way, alongside the production of valuable energy resources and efficient, long-term carbon storage.

Although gas EOR is a matured technology, significant challenges are still faced to improve sweep efficiencies.^9^ Due to the high mobility of gases and the complex porous structure of reservoirs, complications such as early breakthrough, viscous fingering, and gravity segregation occur. Additionally, the gas tends to move through the high permeable zones leaving the tighter zones unswept.^10^ Water alternating gas injection and co-injection with the aqueous phase have been proposed to handle the difficulties. These processes form foams that significantly decrease gas mobility and improve oil recovery.^11^ Foams are discontinuous gas phases trapped by continuous thin aqueous films. Having higher viscosities than gases, they can displace oil more efficiently. In addition, by blocking high permeable pores, foams divert the displaced fluid to unswept pores, improving sweep efficiency^12^ and the subsurface storability of CO_2_.^13^ However, the complex physics of foams still requires further investigation and research to become fully understood and enable foams to be widely applied. Foams are only kinetically stable, depending on various operational and reservoir parameters.^14–16^ Numerous screening and optimization studies were conducted to achieve foams with desired strength under reservoir conditions. In one of these studies,^17^ various mixtures of anionic surfactants were used at different concentrations and foam qualities to obtain optimum foam strength. The study also showed a substantial increase in oil recovery *via* supercritical CO_2_ foam flooding. Almobarky *et al.* investigated the effect of salinity and foam quality on the mobility of the foam in sandstone formations.^18^ Additionally, the performance of foam flooding has been compared with supercritical CO_2_ injection.^19^ Zeng *et al.* used a mixture of gases (CH_4_, CO_2_, N_2_) and investigated foam mobility control in porous media.^20^ These experimental studies can typically focus on optimizing only a few parameters in a small range, as foam experiments are tedious and costly.

Modeling can alternatively correlate operational and reservoir parameters to the rheological properties of the foam. Currently, available modeling techniques have been compared by Hematpur *et al.* as shown in Table 1.^21^ Empirical modeling approaches are the most common since foam behavior can be easily parameterized. Among them, the CMG-STARS calculates the relative foam permeability using a mobility reduction factor (FM) according to the:

1 *K*^f^_rg_ = *K*_rg_ × FM

2
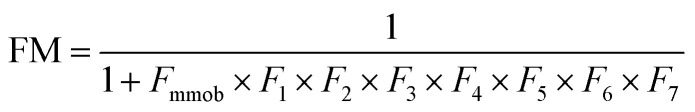
where *K*_rg_ is the gas relative permeability, *K*^f^_rg_ is the gas relative permeability in the presence of foam, and *F*_mmob_ is the maximum capacity of foam mobility reduction. Finally, the various *F*_*i*_ parameters represent the effects of surfactant concentration, oil saturation, injection velocity, capillary pressure, oil composition, salinity, and water saturation. All these *F*_*i*_ parameters are estimated *via* empirical equations, the parameters of which are fitted on experimental and simulation data. Additionally, it is challenging for conventional modeling techniques to consider the effect of several important reservoir parameters, such as temperature and pressure.^21–23^ In such cases, re-tuning of the model parameters is typically required.

**Table 1:** Comparison of foam modeling approaches^21^

Categories	Number of model parameters	Parameter fitting difficulty	Time consumption for simulation	Usage frequency
Empirical approaches	A large number of parameters	Relatively easy	Shorter time	Widely used
Mechanistic approaches	A few parameters	Difficult	Long time	Rarely used

During the last decade, the application of machine learning (ML) to EOR has gained attention. ML algorithms can discover relationships between input parameters and targeted quantities based on experimental studies and provide predictions for unknown situations. Such algorithms have been applied in various studies, such as predicting the most efficient EOR approach under specific conditions or tuning the operational parameters of a particular process of EOR.^24–35^ Recently, studies were also carried out to predict apparent foam viscosity, one of the most important rheological properties of foams, that can be described as viscosity at a given shear rate. Olukoga and Feng have used ML models to provide predictions for nanoparticle-stabilized CO_2_ foam in the bulk phase.^36^ Experimentally obtained rheology data were used to train the ML models having nanoparticle concentration, shear rate, foam quality, salinity, and temperature as input parameters. Various algorithms were used to establish predictive models and estimate each parameter's relative importance. However, no explicit discussion on the effect of input parameters from the physical standpoint was provided. Additionally, the study was limited to a bulk phase study, and no application to porous media was included. Similarly, Ahmed *et al.* used a deep-learning approach for modeling surfactant-stabilized foam in bulk media.^37^ The authors developed a 6-parameter model considering pressure addition to Olukoga and Feng.^36^ The studies confirmed that ML algorithms are a fast and robust methodology that can predict the behavior of complex fluids like foam. At the same time, conventional modeling techniques require too much effort and fail to address some significant reservoir parameters. Though previous studies show the potential of data-driven approaches to estimate foam rheological properties, they were typically limited to data from single research and only in bulk media. Although applying foams for EOR and carbon sequestration porous media is essential, no systematic porous media rheology studies with ML models have yet been performed in the literature.

In the present work, we used a dataset from various experimental studies to develop predictive models for the surfactant-stabilized CO_2_ apparent foam viscosity in bulk and porous media at sandstone formations. We have deployed six different ML algorithms to construct predictive models and thoroughly evaluated their accuracy. Absolute permeability, Darcy velocity, surfactant concentration, salinity, foam quality, temperature, and pressure are selected as parameters of the model for porous media calculations. After successfully building an ML-based model for apparent viscosity at oil-free sandstone formations, predictions were made for optimum foam quality at different injection rates of foam. This will enable us to obtain the conditions for the highest apparent viscosity that can yield both maximum oil recovery and the lowest mobility for improved CO_2_ utilization and storage.

## 2. Methodology

### 2.1 Experimental data

Experimental data on the CO_2_ apparent foam viscosity under the flow loop were selected from various sources,^38–42^ creating a dataset containing 157 examples. In all experiments, a mixture of alpha olefin sulfonate (AOS) surfactant and cocamidopropyl betaine was used. It should be mentioned that the same equipment and experimental setup were used in all selected experiments. At the same time, the dependence of the apparent foam viscosity on the six physical quantities tabulated in Table 2 was examined. A detailed description of all data used and their sources is provided in Table S1.†

**Table 2:** Input parameters for CO_2_ foam in the bulk phase

Parameters	Range
Shear rate (s^−1^)	10–500
Temperature (°C)	40–120
Pressure (MPa)	7–17.3
Salinity (wt%)	0.5–8
Surfactant concentration (wt%)	0.25–1
Foam quality (%)	50–90

For the study of porous media, 145 data points were collected from nine different published works.^17–20,43–47^ In all experiments, the AOS surfactant stabilized CO_2_ foam in sandstone reservoirs. The parameters considered for the ML model are summarized in Table 3, while additional details are provided in Table S2.†

**Table 3:** Input parameters for CO_2_ foam in oil-free sandstone cores

Parameters	Range
Total Darcy velocity (ft per day)	0.6–19
Temperature (°C)	25–80
Pressure (MPa)	2.1–27
Salinity (wt%)	1–15
Surfactant concentration (wt%)	0.5–5
Foam quality (%)	10–98
Permeability (D)	0.1–8.9

### 2.2 Description of affecting parameters

Below, we provide a short description of the physical quantities considered in all experiments (Tables 2 and 3), and we briefly discuss how they qualitatively affect the apparent foam viscosity. These physical quantities were used as inputs (a.k.a. descriptors) by the employed ML algorithms.

#### 2.2.1 Surfactant concentration

The choice of surfactant plays a crucial role in the formation and stability of the foam. It affects the capillary pressure and the interfacial forces between gas and liquid. Usually, surfactants employed for foam formation consist of hydrophilic heads and hydrophobic tails. Depending on the charge of the head, surfactants can be classified into 4 categories: anionic (negative charge), cationic (positive charge), zwitterionic (both charges), and nonionic (no charge).^48^ The performance of a surfactant is highly affected by the conditions of the reservoir and the charges on the rocks. Since sandstone formations are negatively charged, anionic surfactants are usually preferred to avoid material loss. In the current work, AOS surfactant was selected as the foaming agent, and the surfactant concentration (*C*_s_) range investigated was 0.25–1 wt%. It has been observed experimentally that higher surfactant concentrations enhance foam viscosity.^49,50^ Meanwhile, the effect of *C*_s_ on foam behavior becomes relatively insignificant above the critical micelle concentration (CMC).^51^

#### 2.2.2 Foam quality

Foam quality (*F*_q_) is defined as the gas fraction of the foam. Increasing foam quality up to a certain value increases the viscosity significantly. It has been shown that foam viscosity increases to foam quality of 0.9. However, above a threshold *F*_q_ value (∼0.95), foams become too dry to be sustainable, and apparent viscosity decreases sharply.^52^ The foam quality value where the maximum apparent viscosity is observed is called critical foam quality. This is the optimum ratio of gas and aqueous phase to obtain the lowest mobility with the highest injected CO_2_ amount. The foam quality range investigated here was 0.5–0.9.

#### 2.2.3 Temperature

The temperature (*T*) of the reservoir is a significant factor that must be considered for EOR. The foam system should be designed to withstand operational temperature conditions since higher temperatures may destabilize foam and degrade surfactant.^50^ AOS has been experimentally studied for temperatures up to ∼120 °C. It has been seen that destabilizing effect at high temperatures could be compensated by increasing the concentration of surfactant.^17,20,43,46,47,53,54^ The temperature range investigated here was 40–120 °C.

#### 2.2.4 Pressure

Change in pressure (*P*) causes smaller alterations to apparent foam viscosity than the temperature. However, the behavior of CO_2_ foam can significantly change if CO_2_ undergoes a phase change from gas to the supercritical phase due to pressure change.^18^ Accordingly, the pressure was included in the parameters set investigated in this work, and the studied range was 70–173 bar.

#### 2.2.5 Salinity

The high salinity of the aqueous phase harms foam viscosity since it alters the repulsive forces between charged head groups of the surfactant molecules, affecting the surface tension of the aqueous phase and the gas–liquid interactions.^15,50^ According to Majeed *et al.*, surfactant concentrations slightly above CMC are sufficient to compensate for this negative effect.^51^ Experimental studies revealed that under these conditions, AOS has a high tolerance towards salinity due to the presence of Na^+^ cations in the AOS molecule.^51^ Therefore, adding more cations does not notably affect the performance of the surfactant. On the other hand, for surfactant concentration lower than the CMC, an excess number of electrolytes surrounds the negatively charged head groups preventing surfactant molecules to form a micellar structure (foam lamellae). The salinity range investigated here was 0.5–8 wt%.

#### 2.2.6 Shear rate

The shear rate is one of the most important parameters of the foam. It highly depends on the injection rate and describes how fast foam layers move on top of each other. By definition, the shear rate is inversely proportional to apparent viscosity. Therefore, elevated shear rate values noticeable decrease foam apparent viscosity.^54,55^ The shear rate range investigated here based on the experimental data is 10–500 s^−1^.

### 2.3 ML algorithms

We used Python coding to deploy the ML predictive models for the CO_2_ foam apparent viscosity. In what follows, six well-established ML algorithms were used, *i.e.*, decision trees (DT),^56^ random forest (RF),^57^ extremely randomized trees (ERT),^58^ gradient boosting (GB),^59^ extreme gradient boosting (XGB),^60^ and artificial neural network (ANN).^61^ A short description of these algorithms is provided in ESI.† In supervised learning, ML algorithms are trained using labeled examples (training data). Each example consists of several input variables (a.k.a. descriptors or features) and an output (a.k.a. target). Based on the provided training examples, the algorithm correlates descriptors to targets. The obtained model is then used to predict unseen data (test data). The performance of the ML algorithms should be carefully assessed to avoid unreasonable predictions. Usually, a part of the available data is randomly selected and used for the training of the ML algorithm, while the remaining data serve for evaluating its performance. Overfitting represents a challenge for constructing reliable predictive models. It occurs when an ML algorithm accurately reproduces the training data but provides poor predictions on new, unseen cases. To mitigate overfitting, we have employed the *k*-fold cross-validation (*k*-fold CV)^62^ approach with *k* = 10. According to this approach, the training data are divided into *k* subsets (usually *k* = 5 or 10). *k* − 1 subsets are used for the training of the ML algorithm, while the remaining one is used to evaluate its performance. After *k* repetitions of the procedure, all subsets are eventually used for validation. The predictive model that provides the highest accuracy on the validation subset is considered the best-performing one. It should be noted that during this procedure, the most important hyper-parameters of each ML algorithm (see discussion below and ESI†) were optimized. To avoid any bias, the procedure was repeated 100 times using different random training/test data split, and the reported results are the average of the 100 individual runs.

The accuracy of all algorithms was evaluated according to the following statistical metrics: *r*-squared (*R*^2^), mean absolute error (MAE), root mean squared error (RMSE), and weighted average percentage error (WAPE):3
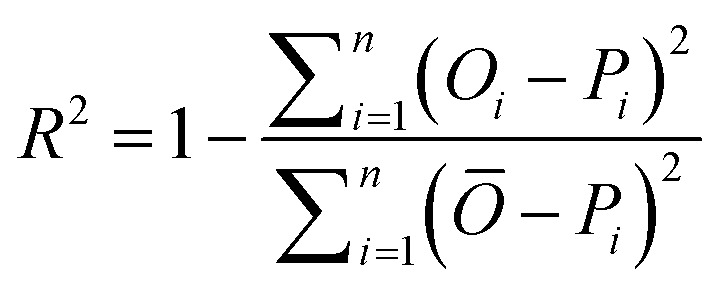
4
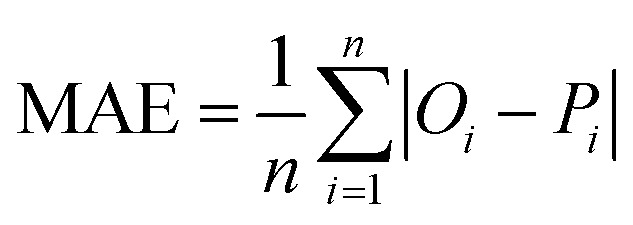
5
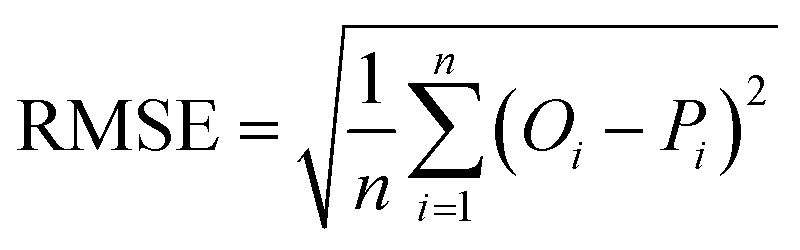
6
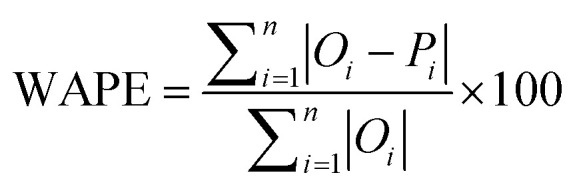


In the expressions above, *O*_*i*_ represents the reference (exact) value of the *i*-th example (out of the total *n* ones), while *P*_*i*_ is the corresponding prediction of the ML algorithm. Finally, *Ō* is the average of all reference values. It should be noticed that as predictions are improving, *R*^2^ value increases (up to the maximum value of 1) while MAE, RMSE and WAPE decrease (up to a minimum value of 0).

## 3. Results and discussion

### 3.1 Bulk foam apparent viscosity

**Fig. 1:**
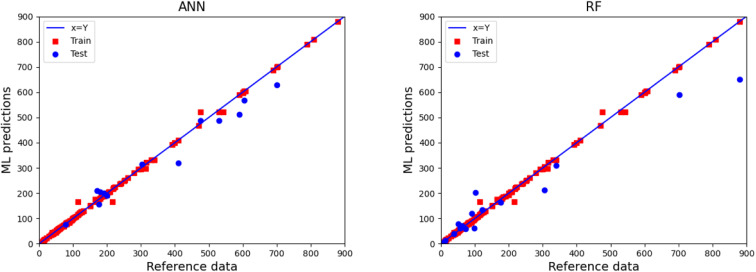
Comparison of the reference values with the predicted ones by the ANN (left panel) and the RF (right panel) algorithms applied to predict bulk foam apparent viscosity. The *x* = *y* diagonal line represents the ideal case.

For most calculations, 90% of the total 157 examples in the database were used to train the ML algorithms, totaling 141 examples, while the remaining 16 examples were used to assess the model's predictive performance. The *k*-fold CV method with *k* = 10 folds was used to construct the ML models and tune each algorithm's most important hyper-parameters. The results of this procedure for the ANN and RF algorithms are illustrated in Fig. 1. Each point represents an example in the database, while red and blue colors correspond to the examples used for the training and testing of the ML algorithms, respectively. The *x*- and *y*-component of each point corresponds to the reference value and to the ML predicted one, respectively. Therefore, the closer a point is to the diagonal line, the better the prediction of the ML algorithm is for this case. As can be seen, the obtained predictions by both the ANN and RF algorithms for the test data (blue points) are considered reasonable.

For a thorough evaluation of the applied ML predictive models, the previous procedure was repeated 100 times, using different randomly selected training and testing sets. The four statistical metrics (*R*^2^, MAE, RMSE, and WAPE) were computed each time, and their average values from the 100 runs are reported in Table 4 for the training and test data. It is evident that the simplest DT algorithm provides the poorest predictions. The RF and the ERT methods provide slightly more accurate results, while a significant improvement is observed when the GB and XGB algorithms are employed. Finally, the most significant improvement is observed in the predictions by the ANN method. For example, the WAPE of the test data by the ANN is almost three times lower than those of the GB and XGB algorithms.

**Table 4:** Accuracy of the applied ML algorithms for the training and testing data in bulk conditions

ML algorithms	*R* ^2^		MAE		RMSE		WAPE	
	Train	Test	Train	Test	Train	Test	Train	Test
DT	0.93	0.67	21.1	51.5	42.0	88.6	12.4	29.3
RF	0.95	0.74	20.7	44.3	39.6	77.5	12.1	25.5
ERT	0.95	0.74	15.0	41.4	30.3	74.4	8.8	23.8
GB	0.99	0.88	2.7	29.7	7.6	52.1	1.6	17.1
XGB	0.99	0.87	4.1	29.6	8.6	52.7	2.4	17.1
ANN	0.99	0.96	5.22	10.9	9.43	18.9	3.06	6.4

**Fig. 2:**
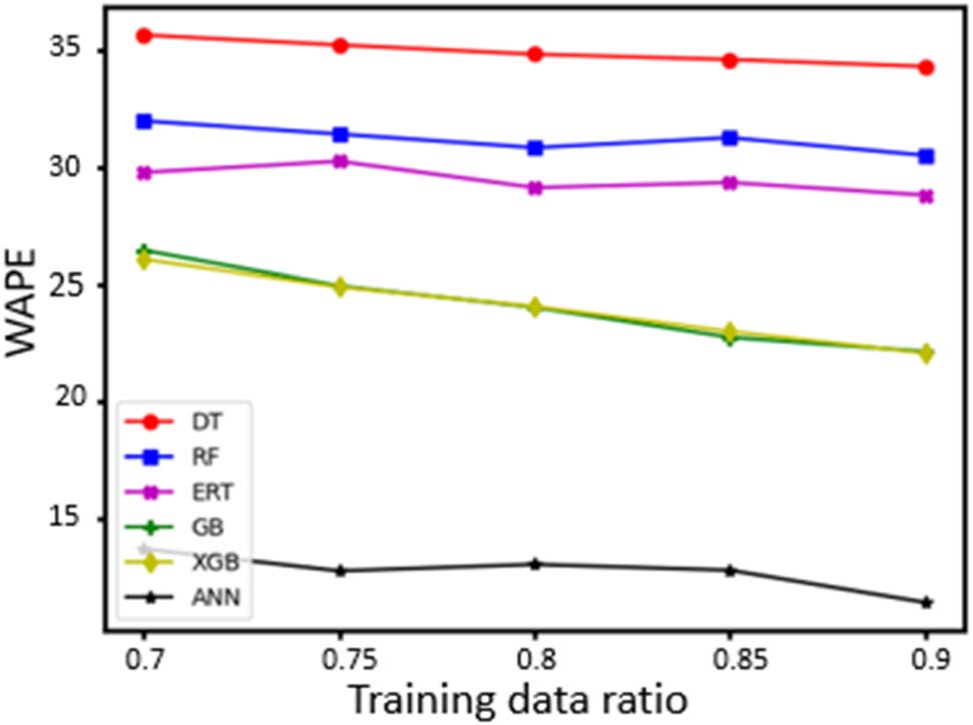
The effect of the training dataset size (110–141 examples) on the WAPE of the tested data.

Notably, the size of the dataset used for constructing the predictive models can be considered relatively small, which may impact the predictions.^63^ Therefore, the effect of the training set size was further investigated. More specifically, set portions between 0.7 and 0.9 (110 and 141 examples, respectively) were used for training the algorithms and the remaining ones for testing. The same protocol as the one described above was employed during these calculations. In Fig. 2, the dependence of WAPE on the training set size is illustrated for all algorithms examined. Evidently, the ANN algorithm outperforms all other employed algorithms for all training set sizes. As expected, improvements in the accuracy of the ML algorithms are observed for increased training set sizes, though they are usually relatively small. Hence, additional experimental data for the training of the ML algorithms would lead to more accurate predictive models.^63^

**Fig. 3:**
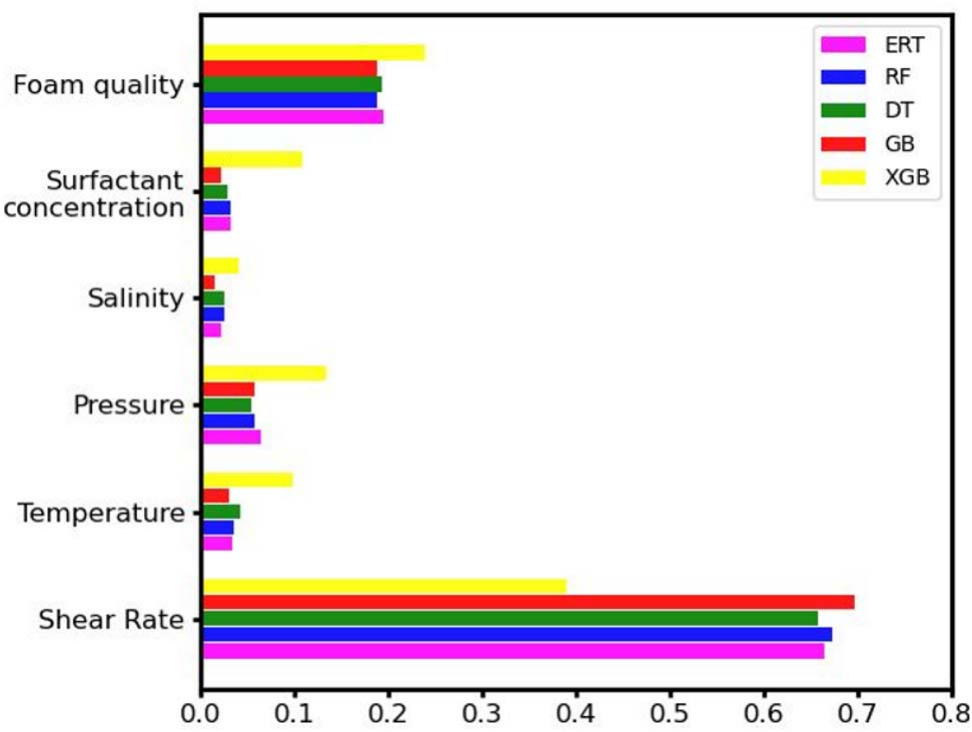
Comparative analysis of the importance of different input parameters.

To quantify the contribution of each physical property to the final model, we performed a comparative analysis of the importance of all inputs used; namely, we assigned a score to all variables based on how useful they are in the prediction of the apparent foam viscosity.^64^ To provide unbiased results, we considered the average of 100 independent runs. The results for all models are summarized in Fig. 3. All ML models show that the shear rate is the most dominant factor for viscosity predictions, as it is inversely proportional to the viscosity *i.e.*, higher shear rates correspond to lower apparent viscosity values.^55^ The foam quality is the second most influential factor in the accuracy of viscosity estimations. According to the ML predictions, the effect of foam quality is still significantly lower than that of the shear rate. In the provided data, the values of foam quality are in the range of 0.5–0.9, namely, lower than that of the critical foam quality.^52^ Outside this region, *e.g.* for *F*_q_ > 0.95, the foam properties are expected to be significantly altered, thus changing the *F*_q_ relative importance accordingly. The remaining parameters are observed to have a lower impact on the final model predictions. Since all selected experimental studies have been carried out with surfactant concentrations well above the CMC (∼0.1 wt%), salinity and surfactant concentration are not expected to noticeably influence the foam apparent viscosity.^49^ Indeed, the applied ML models predict that the influence of these properties is significantly lower than that of foam quality and shear rate. As mentioned in the methodology section, the pressure would influence the behavior of CO_2_ foam only if the latter undergoes a gas to the supercritical phase transition. Since CO_2_ remains in the supercritical phase during all experiments in the database, pressure does not significantly alter the behavior of the foam. Similarly, the temperature has a relatively small contribution to the model predictions, which can be attributed to the high stability of the AOS surfactant within the applied temperature range of 40 to 120 °C and the compensation effects of the high surfactant concentrations in our dataset.^46,47^ Additionally, the aqueous phase contains a stabilizing additive (cocamidopropyl betaine) which significantly enhances foam stability and preserves its apparent viscosity at high temperatures.^39^

### 3.2 Foam apparent viscosity in porous media

**Fig. 4:**
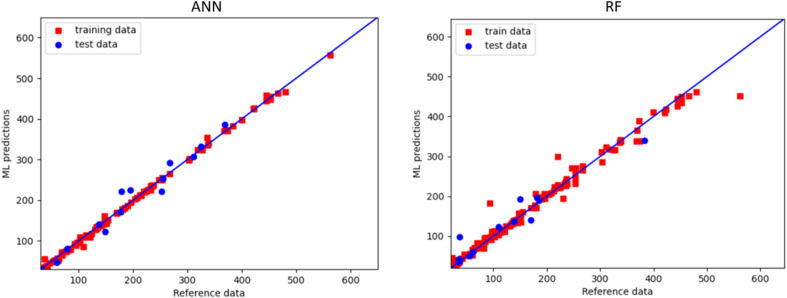
Comparison of the reference values with the predicted ones by the ANN (left panel) and the RF (right panel) algorithms applied to predict foam apparent viscosity in porous media. The *x* = *y* diagonal line represents the ideal case.

ML models were subsequently constructed for predictions of the CO_2_ foam apparent viscosity in porous media using a similar approach. 90% of experimental data were used for training and tuning the hyperparameters of the 6 ML algorithms, while the remaining data were used to test the accuracy. Fig. 4 represents the comparison of predictions and reference values for the ANN and the RF algorithms. It should be noticed that, for the ANN model, all points are very close to the diagonal line that represents the maximum accuracy.^64^ In other words, the model can provide reliable predictions for unseen cases.

A detailed comparison of the performance of all ML algorithms employed is provided in Table 5. Similar to the foam apparent viscosity in bulk, ANN found to provide the most accurate predictions for the foam apparent viscosity in porous media compared to the tree-based approaches. For example, *R*^2^ = 0.93 for ANN and *R*^2^ = 0.80 for XGB were obtained. Therefore, in the next stage, the ANN model will be employed for providing further predictions and optimizing the operational parameters of the CO_2_ foam in porous media.

**Table 5:** Accuracy of the applied ML algorithms for the training and test data in porous media

ML algorithms	*R* ^2^		MAE		RMSE		WAPE	
	Train	Test	Train	Test	Train	Test	Train	Test
DT	1.00	0.69	0.00	31.07	0.00	54.94	0.00	20.01
RF	0.98	0.78	10.01	26.55	19.09	45.84	5.91	17.18
ERT	0.99	0.76	0.01	25.57	0.01	47.61	0.01	16.39
GB	0.99	0.79	8.88	26.63	12.69	44.38	5.24	17.02
XGB	0.99	0.80	0.12	25.06	0.16	44.39	0.07	16.12
ANN	0.99	0.93	3.59	19.38	5.14	28.16	2.16	10.41

**Fig. 5:**
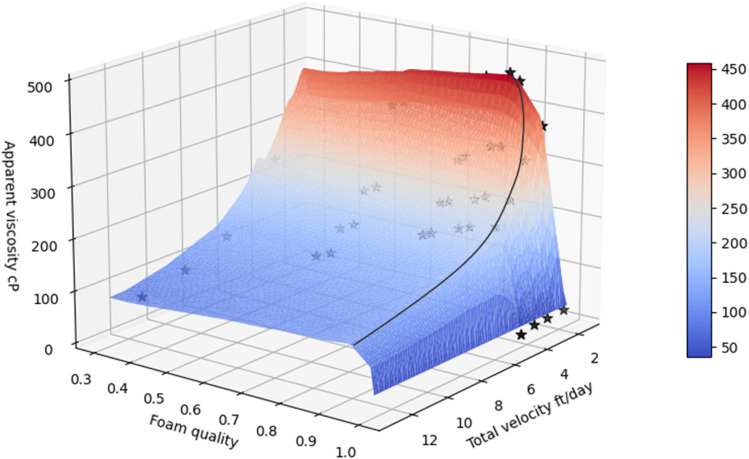
Apparent foam viscosity in porous media for continuous injection velocity and foam quality for fixed temperature (80 °C), pressure (27 MPa), salinity (5.5 wt%), surfactant concentration (1 wt%), permeability = 2.83 D, black line represent the critical foam quality. Filled stars represent literature viscosity data at the given conditions.

One of the advantages of ML-assisted models is that they can provide results for any set of input parameters, given that all parameters lie within their corresponding value range in the training set.^65^ This enables quick and accurate predictions of the CO_2_ foam viscosity without the need of costly experiments or time-consuming conventional modeling techniques. In Fig. 5, a 3D diagram of the viscosity as a function of the foam quality and injection rate is generated for foam at 27 MPa of pressure, 80 °C temperature, 5.5% salinity, and 2.83 D core permeability. The previously developed ANN model was employed for these calculations. The profile can also be used for estimating the critical foam quality for given operational and reservoir conditions. By employing the previous ML predictive models, analysis can be made to obtain the rheological properties that maximize oil recovery and carbon storage. As it can be seen from the Fig. 5 critical foam quality of the CO_2_ foam varies between 0.86 and 0.93 with different injection velocities at the selected conditions. Increasing foam quality further causes the apparent foam viscosity to decrease sharply.^52^ In the same figure, experimental results from literature^47^ are illustrated with star symbols at various conditions. These results were not used during the training of the ML algorithm. It is seen that the ML predictions are close to these results, demonstrating clearly the predictive accuracy of the developed model.

## 4. Conclusions

ML algorithms were utilized to construct predictive models of CO_2_ foam apparent viscosity in bulk and porous media for EOR application and carbon sequestration. Previously reported experimental data were used for the training and evaluation of the algorithms. Based on the obtained results, it can be concluded that ML algorithms can successfully correlate operational parameters to rheological properties, providing reliable predictions in a fraction of the time needed by conventional experimental approaches. In particular, the ANN model provides the most accurate predictions compared to the other tree-based ML algorithms. Although a relatively small dataset was employed, constructing reliable ML models was still possible. Furthermore, by investigating the relative importance of different physical parameters, it was concluded that the most influential factors in predicting foam apparent viscosity in bulk media are the shear rate and the foam quality. After successfully implementing different ML approaches to address the foam rheological properties in flow loop experiments, the behavior of CO_2_ foam in porous media under reservoir conditions was also examined. After a thorough evaluation of the predictive models performance, the most accurate one (ANN) was used for a more detailed study and visualization of the physical behavior of the apparent foam viscosity and the critical foam quality in porous media as a function of the related parameters. Overall, the data-driven approach employed in this work delivered promising results for predicting key rheological properties of CO_2_ foam in the absence of oil.

## Conflicts of interest

There are no conflicts to declare.

## Supplementary Material

RA-012-D2RA05841C-s001
