# Supplementary material for: Data-driven prediction of in situ CO2 foam strength for enhanced oil recovery and carbon sequestration
Source: RSC Adv. 2022 Dec 14;12(55):35703–11. doi: 10.1039/d2ra05841c (PMC9749141; doi:10.1039/d2ra05841c)
Supplement: RA-012-D2RA05841C-s001 [file RA-012-D2RA05841C-s001.pdf]

Electronic Supplementary Material (ESI) for RSC Advances.
This journal is © The Royal Society of Chemistry 2022

Supporting Information

Data-Driven Prediction of In-Situ CO_2 Foam Strength for Enhanced Oil Recovery and Carbon Sequestration

Javad Iskandarov[1,2], *George S. Fanourgakis*[3], *Shehzad Ahmed*[4], *Waleed Alameri*[4], *George E. Froudakis*[3]*, *Georgios N. Karanikolos*[1,2,5,6]*

[1]Department of Chemical Engineering, Khalifa University, P.O. Box 127788, Abu Dhabi, UAE

[2]Research and Innovation Center on CO_2 and H_2 (RICH), Khalifa University, P.O. Box 127788, Abu Dhabi, UAE

[3]Department of Chemistry, University of Crete, Voutes Campus, GR-70013, Heraklion, Crete, Greece

[4]Department of Petroleum Engineering, Khalifa University, P.O. Box 127788, Abu Dhabi, UAE

[5]Center for Catalysis and Separations (CeCaS), Khalifa University, P.O. Box 127788, Abu Dhabi, UAE

[6]Department of Chemical Engineering, University of Patras, 26504 Patras, Greece

*Corresponding author Emails: frudakis@uoc.gr, georgios.karanikolos@ku.ac.ae

Datasets

Details of the various data and their sources that were used in this study:

Table S1. Bulk foam data

Data points	Shear Rate (1/s)	T (°C)	P (bar)	Salinity (wt%)	C_s (wt%)	Fq (%)	Reference
35	10-500	80	103	0.5-8	0.25-1	80	1
56	10-500	40-120	70-173	3	0.5	80	2
19	10-500	80	103	3	0.5	52-85	3
15	100-500	80	103	3	0.25-1	70-80	4
33	10-250	80	103	3	0.25-1	50-90	5

Table S2. Porous media data

Data points	Permeability (D)	T (C)	P (MPa)	Salinity (wt%)	Fg (%)	Cs (wt%)	u Darcy (ft/day)	Reference
12	0.25	65.6	8.3-16.6	2-15	50-80	1-5.	10	6
10	17.1	50	6.55	3-9	70-90	0.5	1.5	7
20	8.89	20	2.5	2	35-93	0.5-1	1.76-6.4	8
5	0.82	40	13	3.5	70	0.5	0.6-19	9
12	2.3	25	2.1	3	40	98	1-4	10
24	1.8	25	9	2	10-99.	0.5	8.46-16.92	11
13	2.056	40	9	1	10-92	0.5	1.5	12
13	0.1	50	13.8	3	80-95	0.5	4.1	13
36	2.83	80	27	5.5	35-100	0.5	1.7-12.1	14

Algorithms

We provide below a brief description of the ML algorithms employed in the present study, along with the most important hyper-parameters of each algorithm that were optimized during the algorithm training.

Decision Tree (DT) algorithm:

The DT algorithm is one of the simplest ML algorithms used for classification and regression problems. During training, the DT algorithm forms a tree with root, decision and leaf nodes. Based on the descriptor's values, the algorithm continuously divides input data at decision nodes into subsets until no individual samples remain. The nodes that don't split into more nodes are called leaf or terminal nodes. In the DT algorithm, the main hyper parameters that need to be optimized are the number of minimum samples split and minimum samples leaf.

Minimum samples split = 2

Minimum samples leaf = 1

Random Forest (RF) algorithm:

The RF algorithm is another tree-based ML algorithm widely adopted for classification and regression tasks. It operates on a specified number of DT which perform the same task, while their results are aggregated into the final prediction. The most important tuned parameters of this algorithm are the number of trees and minimum samples leaf.

Number of trees = 100

Minimum samples split = 2

Minimum samples leaf = 1

Extremely Randomized Tree (ERT) algorithm:

The ERT algorithm is a tree-based approach that has two main differences from the RF algorithm. The first difference is that, in ERT algorithm all trees are trained with the whole training dataset, while in RF different subsets are assigned to the trees. The second difference is that in ERT nodes are split based on random splits, while in RF based on the best split among a random subset of features selected at every node.

Number of trees = 100

Minimum samples split = 2

Minimum samples leaf = 1

Gradient Boost (GB) algorithm:

The GB algorithm is another well-established tree-based algorithm that operates several DT. Unlike the RF algorithm, each tree in GB is built based on the results of the previous one. More specifically, each new tree focuses only on data unassigned by the previous trees. The algorithm uses loss functions, such as the mean squared error (MSE), to identify the unassigned data. The gradient descent method is then employed to minimize the value of the loss function. The samples with the higher errors between predicted and reference values will be assigned to the next tree for further calculations.

Number of trees = 100

Minimum samples split = 2

Minimum samples leaf = 1

Max depth = 10

Extreme Gradient Boost (XGB) algorithm:

The XGB algorithm is a specific implementation of the GB algorithm aiming to increase the accuracy and speed of the latter during training. One of the main changes over GB is that XGB uses second order derivatives of the loss functions enabling in this way its faster and more accurate minimization.

Number of trees = 100

Max depth = 10

Artificial Neural Network (ANN) algorithm:

The ANN algorithm is a computational approach that mimics the human brain. It consists of connected nodes called neurons through which, computational models can be built via tuning specific parameters. Based on its input values each neuron produces a single output which sends to other neurons for further calculations:

$$neuron\ output = input \times weight + bias$$

An ANN consists of the following main three parts:

- The input layer (descriptors)
- One or more hidden layers (calculation neurons)
- The output layer (outputs)

Initially, weights are assigned to each neuron randomly or by empirical approaches. During training, the algorithm multiplies the inputs with the weights of all neurons in the hidden layers and compares the values in the output layer with the reference values. The model is then optimized by properly adjusting the weights of neurons in order to reduce differences between predicted and reference values. Through back propagation the weights are updated reducing the value of a loss function (e.g., RMSE or MAE). The forward and backward propagation

(a.k.a. epoch) is repeated for a maximum number of times (500 in this work) or until the desired accuracy is achieved. In this study, after trial and error, 5 hidden layers with 28 neurons each were selected as the optimum parameters.

Optimizer = Adam

Loss function = MAE

Activation function = Relu

Epochs = 500

References

1 S. Ahmed, K. A. Elraies, M. R. Hashmet and A. S. Hanamertani, Viscosity models for polymer free CO2 foam fracturing fluid with the effect of surfactant concentration, salinity and shear rate, *Energies (Basel)*, , DOI:10.3390/en10121970.

2 S. Ahmed, K. A. Elraies, M. R. Hashmet and M. S. Alnarabiji, Empirical modeling of the viscosity of supercritical carbon dioxide foam fracturing fluid under different downhole conditions, *Energies (Basel)*, , DOI:10.3390/en11040782.

3 S. Ahmed, K. A. Elraies, J. Forooozesh, S. R. Bt Mohd Shafian, M. R. Hashmet, I. C. C. Hsia and A. Almansour, Experimental investigation of immiscible supercritical carbon dioxide foam rheology for improved oil recovery, *Journal of Earth Science*, 2017, **28**, 835–841.

4 S. Ahmed, K. A. Elraies, A. S. Hanamertani, M. R. Hashmet, S. R. Shafian and I. C. Hsia, in *Day 1 Mon, November 11, 2019*, SPE, 2019.

5 S. Ahmed, K. A. Elraies, M. R. Hashmet, A. S. Hanamartani and S. R. Bt Mohd Shafian, in *All Days*, SPE, 2018.

6 Y. Bian and G. Penny, in *All Days*, SPE, 2012.

7 M. A. Almobarky, Z. AlYousif and D. Schechter, Gas/water foams stabilized with a newly developed anionic surfactant for gas mobility control applications, *Pet Sci*, 2020, **17**, 1025–1036.

8 J. Zhao and F. Torabi, Experimental investigation and modelling of CO2 foam flow in heavy oil systems, *Can J Chem Eng*, 2020, **98**, 147–157.

9 G. Batôt, M. Fleury and L. Nabzar, in *Energy Procedia*, Elsevier Ltd, 2017, vol. 114, pp. 4129–4139.

10 Y. Zeng, R. Farajzadeh, A. A. Eftekhari, S. Vincent-Bonnieu, A. Muthuswamy, W. R. Rossen, G. J. Hirasaki and S. L. Biswal, Role of Gas Type on Foam Transport in Porous Media, *Langmuir*, 2016, **32**, 6239–6245.

11 H. T. Horjen, *CO 2 Foam Stabilization with Nanoparticles and EOR in Fractured Carbonate Systems*, 2015.

12 S. Kahrobaei, K. Li, S. Vincent-Bonnieu and R. Farajzadeh, Effects of compositional variations on CO2 foam under miscible conditions, *AIChE Journal*, 2018, **64**, 758–764.

13 M. A. Q. Siddiqui and R. N. Gajbhiye, Stability and texture of CO2/N2 foam in sandstone, *Colloids Surf A Physicochem Eng Asp*, 2017, **534**, 26–37.

14 T. Føyen, Z. P. Alcorn, M. A. Fernø, A. Barrabino and T. Holt, CO2 mobility reduction using foam stabilized by CO2- and water-soluble surfactants, *J Pet Sci Eng*, 2021, **196**, 107651.
